# 1291. PROVE (Retrospective Cefiderocol Chart Review) Study of Real-World Outcomes and Safety in the Treatment of Patients with Gram-negative Bacterial Infections in the US and Europe

**DOI:** 10.1093/ofid/ofab466.1483

**Published:** 2021-12-04

**Authors:** Stephen Marcella, Teena Chopra, Teena Chopra, Jose A Vazquez, Steven Smoke, Ryan K Shields, David van Duin

**Affiliations:** 1 Shionogi Inc., Florham Park, New Jersey; 2 Detroit Medical Center, Wayne State University, Detroit, MI; 3 Medical College of Georgia at Augusta University, Augusta, Georgia; 4 Saint Barnabas Medical Center, Milltown, New Jersey; 5 University of Pittsburgh, Pittsburgh, Pennsylvania; 6 University of North Carolina, Chapel Hill, North Carolina

## Abstract

**Background:**

Gram-negative bacterial resistance is a global health problem. Limited treatment options exist, especially for carbapenem resistant (CR) pathogens containing metallo-β-lactamases (MBLs) and multidrug resistant non-lactose fermenting bacteria. Cefiderocol (CFDC) retains activity against resistant strains. We describe the objectives, design, and early results of PROVE, a real world retrospective study of CFDC use.

**Methods:**

PROVE is a multi-center, chart review study of CFDC use for resistant Gram-negative infections (GNI). Cases were eligible if they received ≥ 72 hrs of CFDC. Demographics, comorbidity, pathogen, infection site, and treatment course were assessed. Outcomes included all-cause 14-day and inpatient mortality and length of stay (LOS). Clinical resolution was defined by documentation that clinical signs and/or symptoms had resolved or improved without relapse.

**Results:**

24 patients who were treated with CFDC at 2 sites were included to date. Median age was 48 years (Range: 19 - 69 years); 33% were female. The most common comorbidity was diabetes (n=7, 29%). Median total ICU LOS was 36 days. Targeted treatment of documented GNI without preceding failure of prior therapy accounted for 71% of CFDC use. Empirical and salvage treatments accounted for 4% and 25% respectively (Table 1). Median time from admission to 1st CFDC dose was 21 days. *Acinetobacter baumannii* and *Pseudomonas aeruginosa* accounted for > 75% of isolates (Fig.1). 92% of patients had CR isolates; > 50% were respiratory. Sensitivity to CFDC was tested in 58% of which 71% were sensitive. All-cause 14-day post-CFDC mortality was 13% (95% CI: 2, 27) and overall hospital mortality 25% (95% CI: 6, 44). Clinical resolution was reached in 54% (95% CI: 33, 76). Median post-CFDC LOS was 40 days. Outcomes were stratified by key covariates (Table 2).

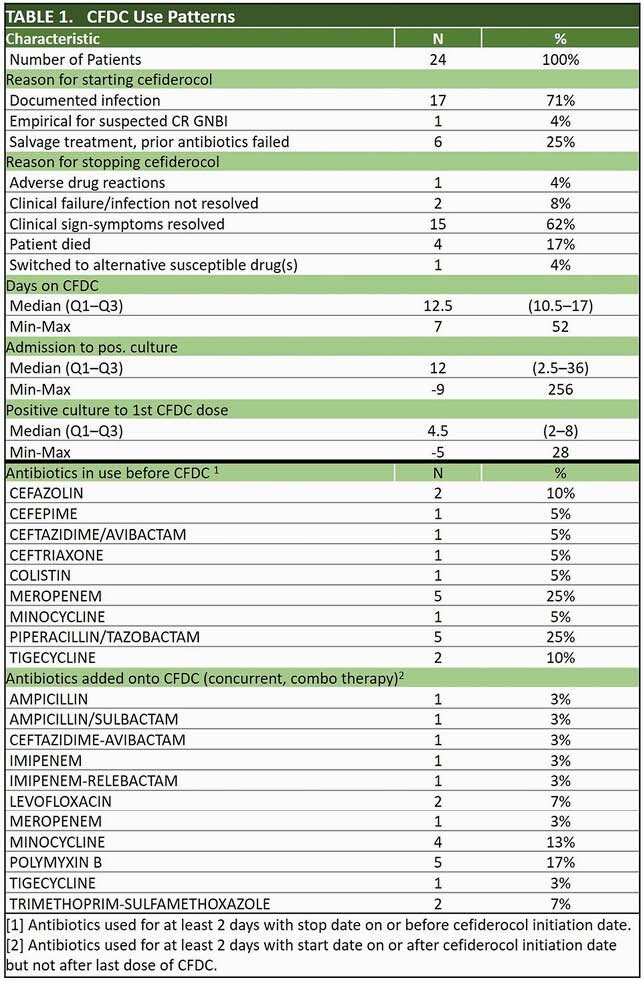

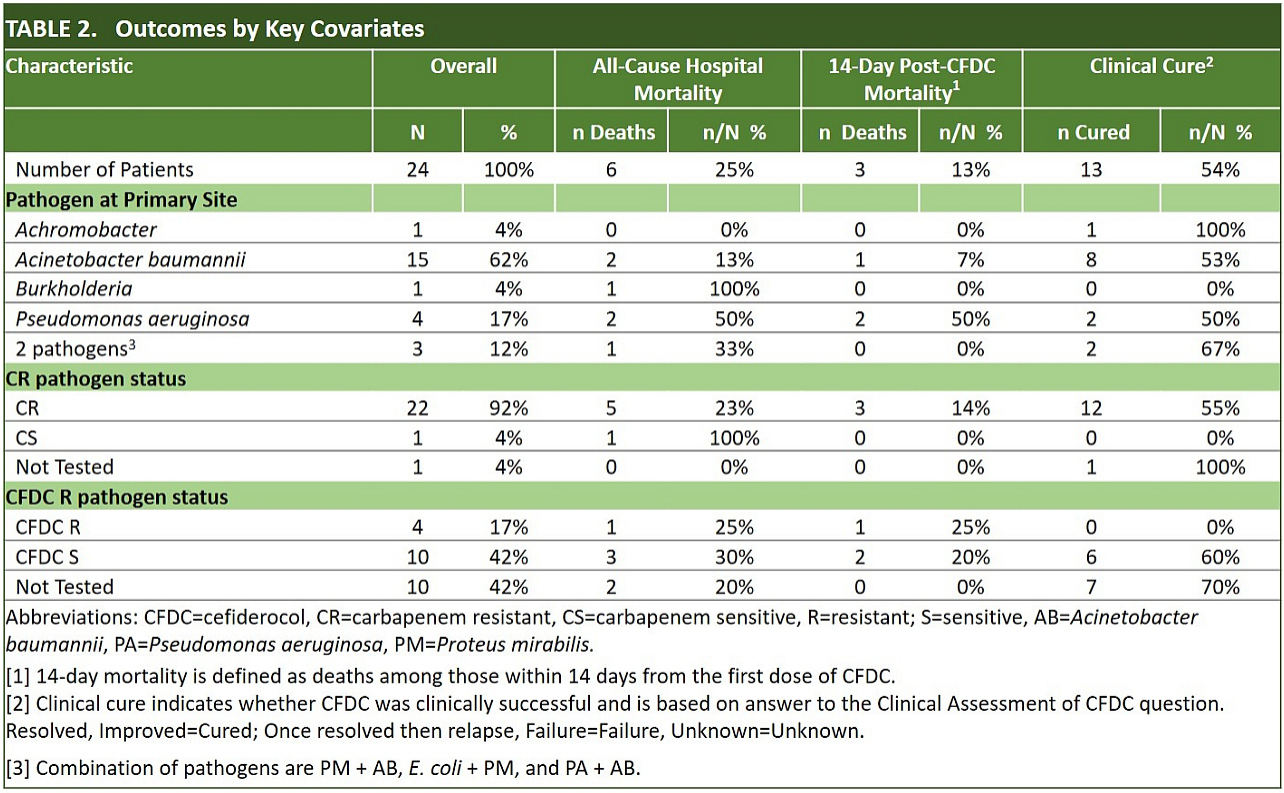

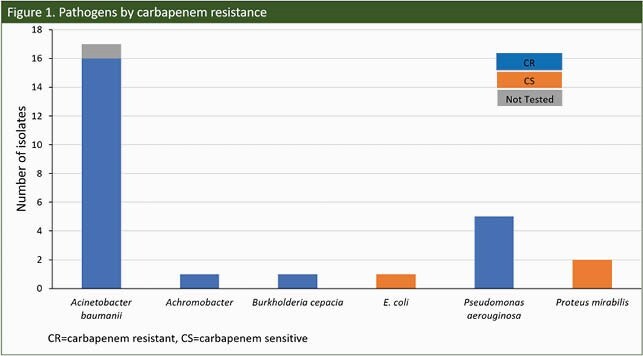

**Conclusion:**

We present initial data for real world use of CFDC for resistant GNI. Patients were complex with multiple comorbidities, some hospitalized for long periods before their index GNI. Outcomes largely reflect this patient population. Additional data are needed to determine the optimal role of CFDC. PROVE offers an opportunity to see how CFDC is being utilized in various settings as well as a first look at key, real world outcomes.

**Disclosures:**

**Stephen Marcella, MD, MPH**, **Shionogi, Inc** (Employee) **Steven Smoke, PharmD**, **Karius** (Advisor or Review Panel member)**Shionogi** (Scientific Research Study Investigator, Advisor or Review Panel member) **Ryan K. Shields, PharmD, MS**, **Shionogi** (Consultant, Research Grant or Support) **David van Duin, MD, PhD**, **Entasis** (Advisor or Review Panel member)**genentech** (Advisor or Review Panel member)**Karius** (Advisor or Review Panel member)**Merck** (Grant/Research Support, Advisor or Review Panel member)**Pfizer** (Consultant, Advisor or Review Panel member)**Qpex** (Advisor or Review Panel member)**Shionogi** (Grant/Research Support, Scientific Research Study Investigator, Advisor or Review Panel member)**Utility** (Advisor or Review Panel member)

